# Sugar-Sweetened Beverage Consumption in Relation to Obesity and Metabolic Syndrome among Korean Adults: A Cross-Sectional Study from the 2012–2016 Korean National Health and Nutrition Examination Survey (KNHANES)

**DOI:** 10.3390/nu10101467

**Published:** 2018-10-09

**Authors:** Sangah Shin, Seong-Ah Kim, Jinwoo Ha, Kyungjoon Lim

**Affiliations:** 1 Department of Food and Nutrition, Chung-Ang University, Gyeonggi-do 17546, Korea; vmffl1@cau.ac.kr; 2 Institute of Health and Environment, Seoul National University, Seoul 08826, Korea; ksacute@snu.ac.kr; 3 Department of Physiology, Anatomy & Microbiology, La Trobe University, Melbourne 3086, Australia; k.lim@latrobe.edu.au

**Keywords:** sugar-sweetened beverage, obesity, metabolic syndrome, Korean, adults

## Abstract

It is well known that the consumption of sugar-sweetened beverages (SSBs) increases the risk of developing obesity and metabolic syndrome (MetS). However, there are not many studies investigating the link between SSBs and increased incidences of diseases in the Asian population, and in particular, in Korea. We explored the association of SSB consumption with the risk of developing obesity and MetS among Korean adults (12,112 participants from the 2012–2016 Korean National Health and Nutrition Examination Survey). We calculated the total SSB consumption frequency by counting each beverage item, including soda beverages, fruit juices, and sweetened rice drinks. Obesity was defined as a body mass index ≥25 kg/m^2^, and MetS was defined using the National Cholesterol Education Program, Adult Treatment Panel III. A survey logistic regression analyses was conducted to examine the association of SSB consumption with obesity and MetS, adjusting for related confounders such as age, energy intake, household income, education, alcohol drinking, smoking status, and physical activity. The SSB consumption was positively associated with an increased risk of the prevalence for obesity (Odd ratio (OR): 1.60; 95% confidence interval (CI): 1.23–2.09; *p* for trend = 0.0009) and MetS (OR: 1.61; 95% CI: 1.20–2.16; *p* for trend = 0.0003) among women. In men, SSB consumption only contributed to a higher prevalence of obesity (OR: 1.38; 95% CI: 1.11–1.72; *p* for trend = 0.0041). In conclusion, increased consumption of SSBs was closely linked with a higher prevalence of obesity and MetS in the Korean population.

## 1. Introduction

It is well known that obesity is one of the main causes of cardiovascular disease (CVD) and metabolic syndrome (MetS) [1]. Obesity is defined as a body mass index (BMI) higher than 25 kg/m^2^ for adults in Asian and Pacific regions according to the International Obesity Task Force [2]. The MetS is characterized by impaired glucose homeostasis, hypertension, reduced high-density lipoprotein cholesterol (HDL-C), and elevated triglyceride [3].

The prevalence of obesity and MetS is increasing globally [4] and in Korea, the prevalence of obesity has continuously increased from 25.7% in 1998 to 37.9% in 2013 among men. Furthermore, cases of extreme obesity (i.e., BMI ≥ 30 kg/m^2^) among women increased from 3.0% in 1998 to 4.7% in 2012 [5]. The prevalence of MetS has also increased from 24.9% in 1998 to 28.9% in 2013 among Korean adults [6,7] and in particular, the prevalence of early onset MetS among children has increased significantly during this period [6].

Importantly, dietary factors are the best-known risk factors for obesity and MetS [8,9]. In particularly, sugar-sweetened beverages (SSBs) such as soft drinks, fruit juices, and sports drinks are considered to be crucial risk factors in the development of obesity and MetS [10,11]. The consumption of SSBs has significantly increased over the past decades and is likely to contribute to the onset of obesity and type 2 diabetes [12,13,14]. In this regard, SSB consumption has been steadily increasing among the Asian population in recent decades, although this is still lower than that among Western [15]. In this regard, several studies have been performed to identify the association between SSB consumption and obesity or cardiometabolic markers among the Western population [14,16,17,18] as well as among Asians [19,20]. For instance, SSB consumption increased the risk of abdominal obesity in Chinese children and adolescents [19] and is linked to a higher risk of MetS among adolescent males in Taiwan [20]. Regarding the Korean population, Ha et al. reported no association between SSB consumption and obesity in adolescent females, while an inverse association was reported in 9–14 years old males [15]. However, most studies conducted in the Asian population focused on children and adolescents, and for this reason, evidence of the association of SSB consumption with obesity or MetS is still limited among Asian adults, particularly in Korean adults.

In Korea, as a result of the rapid economic growth and the adoption of a more Western lifestyle over the past four decades, the food content shifted from a traditional Korean diet which consists primarily with rice or grains and vegetables to diet that are rich in meat, fat, and sweets [21]. A recent Korean National Health and Nutrition Examination Survey (KNHANES) study reported that sweetened beverage consumption has dramatically increased from 58 g/day in 2008 to 101 g/day in 2011, and beverages including soda coffee, and fruit and vegetable drinks are the major source of sugar intake from processed foods among the Korean population [22].

Considering that there has been an increase in the consumption of SSBs, which is closely linked to the increased prevalence of obesity and MetS, it is important to examine the association between the SSB and incidences of obesity and MetS in the Korean population. Hence, the aim of the present study was to verify the association of SSB consumption with obesity and MetS (including its individual components) among Korean adults using a large dataset from 2012–2016 Korean National Health and Nutrition Examination Survey.

## 2. Materials and Methods 

### 2.1. Study Design

The KNHANES is a nationwide cross-sectional survey performed by the Korean Center for Disease Control and Prevention. The KNHANES is comprised of health interview surveys, health examination surveys, and nutrition surveys [23]. Details regarding the study design of KNHANES have been stated on the KNHANES website (http://knhanes.cdc.go.kr/).

The current study included participants aged 35–65 years who completed all three surveys of the 2012–2016 KNHANES. Of the 15,735 participants, 3623 were excluded as a result of missing information on BMI (*n* = 96), MetS components (*n* = 4), or a doubtful energy intake (<500 or >5000 kcal/day; *n* = 3525). A total of 12,112 participants comprising 5308 men and 6804 women were eligible for the analysis.

The present study was conducted according to the guidelines stipulated in the Declaration of Helsinki, and the protocols for KNHANES IV-V involving human subjects were approved by the Korean Centers for Disease Control and Prevention Institutional Review Board (2012-01EXP-01-2C, 2013-07CON-03-4C, and 2013-12EXP-03-5C). All participants wrote the informed consent.

### 2.2. Dietary Assessment

The dish-based semi-quantitative Food Frequency Questionnaire (FFQ) was used to evaluate the intake of 112 food and beverage items during the past year. The frequency of consumption was divided into nine categories: none, once/month, 2–3 times/month, once/week, 2–4 times/week, 4–6 times/week, once/day, 2 times/day, and ≥3 times/day. Portions were categorized as being one of three sizes depending on the type of food: 0.5 portion, 1.0 portion, and 1.5 (or 2.0) portion [24].

In the current study, we calculated SSB consumption frequency by summing each beverage item, including soda beverages (i.e., coke and sprite), fruit juices, and sweetened rice drinks in accordance with previous studies [25]. To categorize SSB consumption, we considered the data distribution of frequency of SSB consumption and previous studies conducted among the Korean population [26]. Consequently, we categorized our subject into four groups across the frequency of consumption of SSBs: non-SSB drinkers, less than 2 times/week, 3–6 times/week, and more than once a day based on SSB consumption frequency by summing each beverage item.

### 2.3. Definition of Obesity and Metabolic Syndrome

All medical examinations, such as blood pressure (BP) measurement, blood component test, and physical measurement, were conducted by trained medical staffs in accordance with standardized protocol. BP was measured using a standard mercury sphygmomanometer (Baumanometer; Baum Co., Inc., Copiague, NY, USA) with the participants in a sitting position after participants had rested for at least 10 min. In all participants, the BP was measured on two separate occasions at 5 min intervals in a quiet atmosphere. The mean of these two measurements was used in the current study. Blood samples were collected from the antecubital vein after fasting for at least eight hours, were immediately refrigerated, and were transported to low-temperature storage at the Central Testing Institute in Seoul, Korea. Fasting plasma glucose, total cholesterol, triglyceride, and HDL-C levels from all participants were measured enzymatically using a Hitachi Automatic Analyzer 7600-210 (Hitachi, Tokyo, Japan).

Height and body weight were measured to the nearest 0.1 kg and 0.1 cm, respectively, with participants wearing light clothes without shoes. BMI was calculated as weight in kilograms divided by the square of height in meters. Waist circumference was measured to the nearest 0.1 cm in a horizontal plane at the midpoint between the iliac crest and the costal margin. The cut-off value for obesity was defined as a BMI of 25 kg/m^2^ or higher according to the International Obesity Task Force for adults in Asian and Pacific regions [2].

MetS was diagnosed if three or more of the following criteria were met according to the National Cholesterol Education Program Adult Treatment Panel III [27]: (1) abdominal obesity (WC ≥ 90 for men and WC ≥ 80 cm for women); (2) elevated triglycerides (≥150 mg/dL or drug treatment for high triglyceride); (3) reduced HDL-C (≤40 mg/dL for men and ≤50 mg/dL in women); (4) elevated BP (systolic BP ≥130 mmHg or diastolic BP ≥85 mmHg or drug treatment for hypertension); and (5) elevated fasting glucose (≥100 mg/dL or drug treatment for hypoglycemic agent or insulin).

### 2.4. Confounding Variables

Demographic variables such as age, household income, and educational level were acquired from a self-report questionnaire. Educational level was categorized into four: elementary school or lower, middle school, high school, and college or higher. The household income was categorized into quartiles according to sex and age groups. Health-related lifestyle variables included smoking status (never smoker, past smoker, and current smoker), alcohol consumption (non-drinker, moderate drinker, and heavy drinker), and physical activity (PA). Alcohol consumption was assessed by questionnaire about their drinking behavior, including the average amount of drinking and drinking frequency, during the month prior to the interview. The subjects were classified into three groups according to the amount of alcohol consumed: non-drinkers, moderate drinkers (<30 g/day), and heavy drinkers (≥30 g/day) [28]. PA was estimated from the Korean version of the International Physical Activity Questionnaire (IPAQ), on topics including vigorous and moderate intensity activities, and walking [29]. The minutes of PA were then converted into metabolic equivalents (METs) [30] and then we categorized our subject into three group: “low”, “moderate” or “high” on the basis of the IPAQ guidelines [31]. The high group included: (i) those who performed vigorous-intensity activity on at least three days, achieving a minimum total PA of at least 1500 MET-min/week; or (ii) those who engaged in seven or more days of any combination of walking, moderate-intensity, or vigorous-intensity activities, achieving a minimum total PA of at least 3000 MET-min/week. The moderate group comprised individuals who carried out: (i) three or more days of vigorous-intensity activity of at least 20 min/day; (ii) five or more days of moderate-intensity activity and/or walking of at least 30 min/day; or (iii) five or more days of any combination of walking, moderate-intensity, or vigorous intensity activities, achieving a minimum total PA of at least 600 MET-min/week. Individuals who did not meet the criteria for two categories were categorized into the low activity group.

### 2.5. Statistical Analysis

All statistical analyses used sampling weight by the Statistical Analysis System (SAS) survey procedure to consider sampling design of the national survey. We evaluated the sex-specific association between SSB consumption and risk of prevalence for obesity/MetS separately by sex, because sex difference has been suggested to play a role in the risk of chronic diseases including obesity and MetS related to dietary factors [32].

The general characteristics and nutrient intake according to SSB consumption categories were presented as means ± standard error for continuous variables and as percent and standard error of percent for categorical variables. The chi-square analysis was used to compare categorical variables, and survey regression models were used to analyze linear trend in the continuous variables. 

To examine the association of SSB consumption with obesity and MetS, we used the survey logistic regression model, with the non-SSB drinker group as the reference group. All multivariate analyses were adjusted for age (continuous), energy intake (continuous), household income (lowest, lower middle, upper middle, and highest), education level (under elementary school, middle school, high school, and over college), alcohol consumption (non-drinker, moderate drinker, heavy drinker), smoking status (never smoker, past smoker, and current smoker), and physical activity (low, moderate, high). 

All statistical analyses were conducted using SAS 9.4 ver. (SAS Institute Inc., Cary, NC, USA). All *p*-values were two-sided, and *p* < 0.05 was considered statistically significant.

## 3. Results

### 3.1. Patient Characteristics

The prevalence of obesity and MetS was 34.9% (41.8% in men and 26.8% in women) and 26.7% (31.2% in men and 21.5% in women), respectively. The general characteristics of the participants according to SSB consumption are presented in Table 1. The mean age of the non-SSB drinker group was 51.3 ± 0.3 years in men and 47.7 ± 0.3 years in women and that of the group of highest SSB consumption (≥1 serving /day) was 43.2 ± 0.4 year in men and 43.4 ± 0.5 year in women. In both of men and women, mean height and weight were significantly different according to the SSB consumption group (all *p* < 0.05). Men consuming ≥1 serving/day of SSBs had a higher household income (*p* < 0.0001), higher education level (*p* < 0.0001), were less heavy drinkers (*p* = 0.0047), were less current smokers (*p* = 0.0475) when compared with non-SSB drinkers. The group of highest SSB consumption had a higher education level (*p* < 0.0001) and were more heavy drinkers (*p* < 0.0001), were more current smokers (*p* = 0.0212), and had a higher physical activity (*p* = 0.0005) when compared with non-SSB drinkers among women. There were no significant differences for physical activity in men across the SSB consumption groups. Meanwhile, there were no significant differences for household income according to SSB consumption among women. 

### 3.2. Correlation between SSB Consumption and Obesity and Metabolic Syndrome 

Energy intake was significantly increased across SSB consumption in both men and women (*p* = 0.0038 and 0.0082, respectively). SSB consumption was positively associated with carbohydrate intake in men and women (*p* = 0.0021 and 0.0424, respectively) and with fat intake in men (*p* = 0.0058). The percentage of energy from protein and fat significantly increased across SSB consumption levels in men and women (all *p* < 0.0001), whereas the percentage of energy from carbohydrate was negatively associated with SSB consumption in men and women (all *p* < 0.0001) (Table 2).

The BMI and MetS indicators and the prevalence of obesity and MetS (including its individual components) are presented in Table 3. For men, an increased consumption of SSBs was linked to a greater diastolic blood pressure (DBP) (*p* = 0.0107), but was not associated with BMI and other components. Meanwhile, an increased consumption of SSBs was positively associated with BMI, DBP, triglyceride, and fasting glucose among women (*p* = 0.0022, 0.0320, 0.0032, and 0.0070, respectively). There were significant differences for the prevalence of obesity, MetS, elevated BP, reduced HDL-C (only in men), elevated triglyceride (only in women), and elevated fasting glucose (only in men) according to SSB consumption level.

The Odd ratios (ORs) for obesity and MetS (including its individual components) according to SSB consumption by sex are shown in Table 4. SSB consumption was positively associated with the prevalence of obesity, while was not associated with the ORs for MetS (incl. its individual components) according to SSB consumption among men. For women, the SSB consumption was positively associated with obesity, MetS, and all MetS components in women except for elevated BP after adjusting for age, energy intake, household income, education level, alcohol consumption, smoking status, and physical activity. Women in the group of highest SSB consumption (≥1 serving/day) had a 59% higher risk of obesity (Odd ratio (OR): 1.59; 95% confidence interval (CI): 1.22–2.08; *p* for trend = 0.0003) and a 61% higher risk of MetS (OR: 1.61; 95% CI: 1.20–2.16; *p* for trend = 0.0003) compared to women in the non-SSB drinker group. The association of increased waist circumference (*p* for trend = 0.0020), reduced HDL-C (*p* for trend = 0.0154), elevated triglyceride (*p* for trend = 0.0058), and elevated fasting glucose (*p* for trend = 0.0007) with SSB consumption showed a positive linear trend among women after adjusting for potential confounders.

## 4. Discussion

The main findings of the present study indicate that SSB consumption is closely linked to an increased risk of obesity in both men and women after adjustment for potential confounders using data from representative Korean adults. SSB consumption was positively associated with the prevalence of MetS and its individual components including abdominal obesity, reduced HDL-C, and elevated fasting glucose among the Korean population.

Our finding of an association between SSB consumption and MetS and its individual components among adults supports other findings from the Asian population [25,26,33,34,35]. Furthermore, several studies have examined the association of SSB consumption with weight gain or obesity and MetS in adults. Schulze et al. identified that women who increased their SSB consumption had significantly larger increases in weight (4.20–4.69 kg) and BMI (1.53–1.72 kg/m^2^) than women who maintained a low or a high intake or considerably reduced their SSB consumption [36]. In the Framingham Heart Study, higher SSB intake was significantly associated with a higher incidence of obesity, MetS, and individual MetS components including increased waist circumference, impaired fasting glucose, hypertension, hypertriglyceridemia, and low HDL-C [18]. 

In a similar trend, among Korean women consuming ≥4 servings/week of soft drinks, there was a significantly higher risk of MetS incidence when compared with infrequent consumers [26]. Moreover, after multivariate adjustment, frequent soft drink consumption increased the risk of elevated triglyceride and hypertension when compared with those who rarely consumed or did not consume soft drinks [26]. One cohort study, which included 5251 male and female Koreans aged 40–69 years, found that participants drinking one cup of sweetened carbonated beverage per week, on average, were at a 17% elevated risk of MetS [37]. Another Korean cohort study which included 5775 adults reported that subjects in the highest SSB consumption group had a significantly greater (21%) risk of hypertension than that of the lowest group [25]. 

The association between SSB consumption and the risk of increased incidence of hypertension was stronger (51%) in subjects with BMI ≥ 25 kg/m^2^ [25]. In the Singapore Chinese Health Study, participants consuming ≥2 soft drinks per week had a relative risk of type 2 diabetes of 1.42 (95% CI: 1.25, 1.62) compared with those who rarely consumed soft drinks [33]. Regarding the risk of diabetes, a Thai Cohort Study identified that women consuming SSBs once or more per day have higher risk (2.4 times) of type 2 diabetes mellitus compared with infrequent women consumers at the 8-year follow-up, but there was no association among men [35]. These findings from the Asian population suggest that SSB consumption at levels lower than those reported for the Western population could be associated with the development of chronic diseases in the Asian population.

Meanwhile, current findings are in contrast with the results of several cross-sectional studies conducted among the US population. An analysis of data from the Continuing Survey of Food Intakes by Individuals (CSFII) 1989–1911, CSFII 1994–1998, the National Health and Nutrition Examination Survey (NHANES) 1988–1994, and NHANES 1999–2002 showed no significant association between SSB intake and the risk of obesity after multivariate adjustment [38]. These discrepancies among studies may be partly explained by the differences of general characteristics of the study population. According to a recent meta-analysis of 12 studies (eight cross-sectional and four prospective cohort studies), high SSB consumption was associated with a high risk of MetS [39]. Moreover, higher consumption of artificially sweetened beverages was associated with higher risk of MetS in a pooled analysis of both cross-sectional and prospective studies. However, the result from the pooling analysis of cohort studies showed no association between consumption of SSBs and risk of onset of MetS [39].

Importantly, a number of potential key factors can explain the increased risk of obesity and MetS associated with greater SSB consumption. Frequent consumption of SSBs in a liquid form has been related to an increased risk of weight gain and obesity as liquid forms of food lack satiety effects [40]. The consumption of food in liquid form has been linked to a lower degree of energy compensation than that consumed in solid form, thus promoting the overconsumption of energy [41]. 

Furthermore, most SSBs contain excessive amount of added nutritive sweeteners such as high-fructose corn syrup (the primary sweetener used in SSBs). Various studies suggest that high consumption of added sweeteners may be associated with deleterious effects on the metabolic rate [42], and increases the risk of low HDL-C concentration [43], hypertension [18], insulin resistance [44], and hypertriglyceridemia [45]. Excessive consumption of fructose and sucrose from SSBs increases lipid synthesis in the liver, which leads to increased serum triglyceride, cholesterol concentration [46], and visceral adiposity [47]. Importantly, the sugar content in the SSB is easily absorbed into the blood which increases blood glucose level [48], eventually causing the release of insulin [40]. Nonetheless, the association of SSB consumption with obesity and MetS could be explained by the dietary habit and lifestyle behavior among individuals. 

In the current study, we confirmed that a higher intake of SSB is positively associated with unhealthy lifestyles such as smoking and alcohol consumption among women. Surprisingly, there was no clear association between SSB consumption and increased risk of MetS among men. In this regard, estrogen in women affects the renin-angiotensin system, which enhances fat transport and increases the levels of triglyceride and lipoprotein in the blood, whereas androgen in men has the opposite effect to estrogen [49]. Thus, lipid levels could be differently regulated between men and women. These findings suggest sex differences in the association between dietary factors and metabolic risk.

Our study has several strengths, including the large nationally representative sampling design that provided detailed information allowing for the better control of potential confounders among the Korean population. To the best of our knowledge, this is the first study to examined associations between SSB consumption and obesity and MetS (incl. its individual components) at the same time among Korean adults aged 35–65 years. Limitations of the present study are as follows: firstly, an accurate temporal sequence could not be inferred as a result of the nature of the cross-sectional design. Hence, it is difficult to determine a causal relationship of SSB consumption with the risk of obesity and MetS among Korean adults. Secondly, the KNHANES dietary data from FFQ shows a bias error; thus, there is the possibility of an underestimation of the true consumption of SSBs. Regarding the type of SSB, we were only able to include total SSB consumption as sum of soda drinks, fruit juices, and sweetened rice drinks because of data availability. In addition, we did not differentiate between the artificial non-caloric sweeteners and those containing caloric sugar (e.g., fructose or sucrose) because the FFQ in KNHANES had no information about artificially sweetened beverages. Lastly, there were unmeasured and residual confounding factors, which is a problem with all observational studies. Although there are limitations in the current data, we adjusted for several potential confounders in the analyses. 

## 5. Conclusions

SSB consumption is associated with a significantly higher prevalence of obesity in Korean adults. SSB consumption is positively associated with a higher prevalence of MetS and its components including elevated waist circumference, decreased HDL-C, and elevated fasting glucose among women, independent of lifestyle factors. Our findings indicate that SSB consumption should be monitored continually, the objective of identifying the exact mechanism associated with the consumption of SSBs and an increased risk of obesity and MetS should be set, and well-designed prospective cohort studies or randomized controlled trials are needed to confirm and strengthen our findings.

## Figures and Tables

**Table 1:** General characteristics of participants according to sugar-sweetened beverage consumption.

	Sugar-Sweetened Beverage Consumption				*p*-Value ^1)^
	Non-Drinker	≤2/Week	3–6/Week	≥1/Day	
*Men (n* = *5308)*					
No. of participants	1000 (18.8) ^2)^	2259 (42.6)	1247 (23.5)	802 (15.1)	
Age (years)	51.3 ± 0.3	46.3 ± 0.2	43.4 ± 0.3	43.2 ± 0.4	<0.0001
Height (cm)	169.6 ± 0.2	171.2 ± 0.1	172.5 ± 0.2	172.0 ± 0.2	<0.0001
Weight (kg)	70.5 ± 0.4	72.2 ± 0.3	73.2 ± 0.4	73.9 ± 0.4	<0.0001
Household income ^3)^					
Lowest	127 (12.8)	171 (7.6)	63 (5.1)	42 (5.3)	<0.0001
Lower middle	252 (25.4)	529 (23.6)	283 (22.8)	146 (18.3)	
Upper middle	289 (29.1)	745 (33.2)	421 (33.9)	260 (32.7)	
Highest	326 (32.8)	800 (35.6)	476 (38.3)	348 (43.7)	
Education level					
≤Elementary school	165 (17.9)	189 (9.0)	74 (6.5)	21 (2.9)	<0.0001
Middle school	134 (14.5)	201 (9.6)	79 (6.9)	40 (5.5)	
High school	304 (32.9)	719 (34.2)	395 (34.7)	201 (27.6)	
≥College	320 (34.7)	992 (47.2)	591 (51.9)	466 (64.0)	
Alcohol consumption					
Non-drinker	157 (16.3)	303 (13.0)	143 (11.2)	85 (10.8)	0.0047
Moderate drinker	452 (48.0)	1177 (54.9)	656 (56.4)	433 (57.1)	
Heavy drinker	332 (35.6)	680 (32.0)	375 (32.3)	243 (32.1)	
Smoking status					
Never smoker	139 (14.8)	424 (19.6)	243 (20.7)	153 (20.1)	0.0475
Past smoker	397 (42.2)	856 (39.6)	428 (36.5)	291 (38.2)	
Current smoker	404 (43.0)	880 (40.7)	503 (42.8)	317 (41.7)	
Physical activity					
Low	710 (78.2)	1558 (77.4)	815 (74.7)	511 (73.5)	0.0732
Moderate	86 (11.2)	207 (10.7)	128 (12.5)	74 (10.9)	
High	89 (10.6)	229 (12.0)	138 (12.8)	114 (15.6)	
*Women (n* = *6804)*					
No. of participants	1844 (27.1)	3292 (48.4)	1184 (17.4)	484 (7.1)	
Age (year)	47.7 ± 0.3	44.3 ± 0.2	43.9 ± 0.3	43.4 ± 0.5	<0.0001
Height (cm)	158.0 ± 0.2	158.7 ± 0.1	159.2 ± 0.2	158.8 ± 0.3	<0.0001
Weight (kg)	58.3 ± 0.2	58.2 ± 0.2	59.3 ± 0.3	59.8 ± 0.5	0.0052
Household income					
Lowest	221 (12.0)	253 (7.7)	98 (8.3)	38 (7.9)	0.1008
Lower middle	462 (25.2)	808 (24.7)	266 (22.6)	110 (22.9)	
Upper middle	546 (29.7)	1063 (32.5)	389 (33.0)	140 (29.2)	
Highest	607 (33.1)	1151 (35.1)	425 (36.1)	192 (40.0)	
Education level					
≤Elementary school	259 (14.8)	268 (8.7)	73 (6.6)	30 (6.8)	<0.0001
Middle school	223 (12.7)	276 (8.9)	92 (8.3)	30 (6.8)	
High school	688 (39.3)	1164 (37.6)	448 (40.3)	195 (44.1)	
≥ College	581 (33.2)	1384 (44.8)	498 (44.8)	187 (42.3)	
Alcohol consumption					
Non-drinker	596 (32.5)	842 (24.7)	288 (24.8)	106 (24.2)	<0.0001
Moderate drinker	1102 (62.2)	2145 (69.0)	778 (68.0)	321 (67.2)	
Heavy drinker	99 (5.3)	187 (6.3)	74 (7.2)	33 (8.6)	
Smoking status					
Never smoker	1609 (89.6)	2867 (90.3)	1006 (88.2)	396 (86.1)	0.0212
Past smoker	85 (4.7)	169 (5.3)	62 (5.4)	28 (6.1)	
Current smoker	102 (5.7)	138 (4.3)	72 (6.3)	36 (7.8)	
Physical activity					
Low	1494 (85.6)	2592 (84.5)	920 (83.1)	335 (77.6)	0.0005
Moderate	130 (8.0)	258 (8.8)	90 (9.1)	55 (12.9)	
High	103 (6.3)	196 (6.7)	79 (7.8)	40 (9.5)	

^1)^*p*-values were calculated via survey regression for continuous variables and via the complex sampling chi-square test for categorical variables. ^2)^ Values are presented as mean ± standard error or weighted N (percent). ^3)^ Household income was defined as quartile according to sex and age group.

**Table 2:** Nutrient intake according to sugar-sweetened beverage consumption.

	Sugar-Sweetened Beverage Consumption				*p*-Value ^1)^
	Non-Drinker	≤2/Week	3–6/Week	≥1/Day	
*Men (n* = *5308)*					
Energy (kcal/day)	2023.1 ± 23.2 ^2)^	2255.2 ± 17.6	2527.1 ± 26.3	2709.5 ± 36.9	0.0038
Carbohydrate (g/day) ^3)^	315.4 ± 3.0	348.6 ± 2.5	378.5 ± 3.4	405.3 ± 5.1	0.0021
Protein (g/day)	59.8 ± 0.9	71.1 ± 0.7	81.7 ± 1.2	89.0 ± 1.6	0.0932
Fat (g/day)	32.8 ± 0.7	42.0 ± 0.5	51.6 ± 0.9	56.4 ± 1.2	0.0058
% Energy from					
Carbohydrate (% of Energy)	71.2 ± 0.3	68.6 ± 0.2	66.6 ± 0.2	66.3 ± 0.3	<0.0001
Protein (% of Energy)	13.0 ± 0.1	13.6 ± 0.0	13.9 ± 0.1	14.1 ± 0.1	<0.0001
Fat (% of Energy)	15.7 ± 0.2	17.8 ± 0.1	19.5 ± 0.2	19.6 ± 0.2	<0.0001
*Women (n* = *6804)*					
Energy (kcal/day)	1581.1 ± 15.4	1802.9 ± 12.2	1981.5 ± 25.4	2178.6 ± 45.3	0.0082
Carbohydrate (g/day)	265.2 ± 2.4	295.1 ± 2.0	317.0 ± 3.6	344.4 ± 6.6	0.0424
Protein (g/day)	52.4 ± 0.6	61.4 ± 0.5	68.6 ± 1.1	76.7 ± 1.9	0.8612
Fat (g/day)	30.0 ± 0.5	36.8 ± 0.4	43.0 ± 0.8	48.8 ± 1.6	0.1053
% Energy from					
Carbohydrate (% of Energy)	69.6 ± 0.2	67.8 ± 0.2	66.5 ± 0.3	65.8 ± 0.4	<0.0001
Protein (% of Energy)	13.4 ± 0.1	13.8 ± 0.0	14.0 ± 0.1	14.3 ± 0.1	<0.0001
Fat (% of Energy)	16.9 ± 0.2	18.4 ± 0.1	19.5 ± 0.2	19.9 ± 0.3	<0.0001

^1)^*p*-values were calculated via survey regression adjusted for age. ^2)^ Values are presented adjusted mean ± standard errors. ^3)^ Carbohydrate, protein, and fat intake were adjusted via residual method.

**Table 3:** BMI and metabolic syndrome components according to sugar-sweetened beverage consumption.

	Sugar-Sweetened Beverage Consumption				*p*-Value ^1)^
	Non-Drinker	≤2/Week	3–6/Week	≥1/Day	
*Men (n* = *5,308)*					
BMI ^2)^ (kg/m^2^)	24.5 ± 0.1 ^3)^	24.6 ± 0.1	24.6 ± 0.1	24.9 ± 0.1	0.1307
Waist circumference (cm)	85.8 ± 0.3	85.6 ± 0.2	85.5 ± 0.3	86.1 ± 0.3	0.5930
SBP (mmHg)	121.0 ± 0.5	118.9 ± 0.4	118.6 ± 0.5	119.9 ± 0.6	0.0630
DBP (mmHg)	79.8 ± 0.4	79.6 ± 0.2	79.9 ± 0.3	81.2 ± 0.4	0.0107
HDL cholesterol (mg/dL)	46.6 ± 0.4	47.2 ± 0.3	47.0 ± 0.3	47.2 ± 0.4	0.9359
Triglyceride (mg/dL)	184.5 ± 5.7	171.0 ± 3.4	174.0 ± 4.4	185.2 ± 6.2	0.1426
Fasting glucose (mg/dL)	105.5 ± 0.9	101.6 ± 0.5	101.6 ± 0.8	102.6 ± 1.1	0.2385
Prevalence					
Obesity ^4)^	374 (38.5)	930 (40.8)	521 (41.5)	388 (48.6)	0.0011
MetS	385 (37.3)	701 (29.6)	372 (29.2)	259 (31.9)	0.0007
Increased waist circumference	304 (30.8)	654 (28.7)	348 (26.7)	250 (31.1)	0.1640
Elevated blood pressure	506 (49.3)	946 (38.6)	498 (38.6)	342 (41.9)	<0.0001
Reduced HDL cholesterol	352 (35.3)	690 (30.2)	364 (30.3)	220 (25.8)	0.0024
Elevated triglyceride	441 (45.9)	931 (43.3)	524 (43.8)	358 (46.8)	0.3576
Elevated fasting glucose	503 (48.4)	911 (39.1)	472 (36.4)	309 (39.4)	<0.0001
*Women (n* = *6804)*					
BMI (kg/m^2^)	23.4 ± 0.1	23.1 ± 0.1	23.4 ± 0.1	23.7 ± 0.2	0.0022
Waist circumference (cm)	78.0 ± 0.3	77.4 ± 0.2	77.9 ± 0.3	78.1 ± 0.5	0.0502
SBP (mmHg)	113.5 ± 0.4	111.7 ± 0.3	111.8 ± 0.5	112.0 ± 0.7	0.1283
DBP (mmHg)	73.8 ± 0.3	73.7 ± 0.2	73.7 ± 0.3	74.0 ± 0.5	0.0320
HDL cholesterol (mg/dL)	54.8 ± 0.3	54.9 ± 0.2	54.4 ± 0.4	54.2 ± 0.8	0.0934
Triglyceride (mg/dL)	111.4 ± 2.3	108.0 ± 1.6	116.2 ± 2.8	125.5 ± 13.6	0.0032
Fasting glucose (mg/dL)	96.8 ± 0.6	95.2 ± 0.4	97.1 ± 0.7	99.0 ± 1.7	0.0070
Prevalence					
Obesity	507 (27.0)	864 (25.5)	321 (27.4)	149 (32.3)	0.0493
MetS	460 (23.3)	689 (19.8)	261 (22.3)	108 (23.6)	0.0449
Increased waist circumference	726 (37.9)	1161 (34.4)	446 (37.5)	192 (39.4)	0.0632
Elevated blood pressure	514 (26.3)	732 (21.2)	271 (22.2)	99 (20.5)	0.0024
Reduced HDL cholesterol	785 (43.1)	1321 (41.7)	479 (41.6)	200 (45.1)	0.5691
Elevated triglyceride	344 (18.9)	584 (18.1)	245 (22.9)	83 (19.3)	0.0293
Elevated fasting glucose	462 (24.9)	723 (22.8)	265 (24.8)	130 (27.8)	0.1415

Abbreviations: BMI, body mass index; MetS, metabolic syndrome; SBP, systolic blood pressure; DBP, diastolic blood pressure; HDL, high-density lipoprotein.^1)^ *p*-values were calculated via survey regression adjusted for age. ^2)^ Body mass index was calculated as weight (kg)/height (m)^2^. ^3)^ Values are presented as adjusted mean ± standard errors or weighted N (percent). ^4)^ Obesity was defined as body mass index ≥25 kg/m^2^.

**Table 4:** Multivariate adjusted odds ratios ^1)^ and 95% CI for obesity and metabolic syndrome (including its individual components) according to sugar-sweetened beverage consumption.

	Sugar-Sweetened Beverage Consumption				*p* for Trend ^2)^
	Non-Drinker (Reference)	≤2/Week OR (95% CI)	3–6/Week OR (95% CI)	≥1/Day OR (95% CI)	
*Men (n* = *5308)*					
Obesity	1.00	1.05 (0.88–1.26)	1.04 (0.85–1.28)	1.41 (1.13–1.76)	0.0025
Metabolic syndrome	1.00	0.85 (0.71–1.01)	0.92 (0.75–1.12)	1.07 (0.85–1.34)	0.0989
Increased waist circumference	1.00	0.91 (0.75–1.10)	0.82 (0.66–1.02)	1.02 (0.80–1.31)	0.6749
Elevated blood pressure	1.00	0.81 (0.68–0.98)	0.93 (0.76–1.14)	1.10 (0.88–1.37)	0.0145
Reduced HDL cholesterol	1.00	0.90 (0.75–1.09)	1.00 (0.81–1.24)	0.80 (0.63–1.01)	0.2231
Elevated triglyceride	1.00	0.97 (0.81–1.15)	1.00 (0.82–1.22)	1.15 (0.91–1.45)	0.1026
Elevated fasting glucose	1.00	0.91 (0.76–1.09)	0.94 (0.78–1.14)	1.09 (0.87–1.38)	0.1679
*Women (n* = *6804)*					
Obesity	1.00	1.10 (0.93–1.29)	1.23 (1.02–1.49)	1.59 (1.22–2.08)	0.0003
Metabolic syndrome	1.00	1.13 (0.97–1.32)	1.40 (1.13–1.74)	1.61 (1.20–2.16)	0.0003
Increased waist circumference	1.00	1.05 (0.91–1.22)	1.24 (1.03–1.49)	1.37 (1.08–1.75)	0.0020
Elevated blood pressure	1.00	1.09 (0.93–1.29)	1.25 (0.99–1.56)	1.21 (0.88–1.65)	0.1034
Reduced HDL cholesterol	1.00	1.14 (0.99–1.30)	1.15 (0.96–1.38)	1.40 (1.10–1.78)	0.0154
Elevated triglyceride	1.00	1.16 (0.97–1.39)	1.58 (1.26–1.99)	1.31 (0.95–1.81)	0.0058
Elevated fasting glucose	1.00	1.14 (0.97–1.33)	1.30 (1.06–1.61)	1.62 (1.21–2.19)	0.0007

Abbreviations: HDL, high-density lipoprotein. ^1)^ Adjusted for age (continuous), energy intake (continuous), household income (lowest, lower middle, upper middle, highest), education level (under elementary school, middle school, high school, over college), alcohol consumption (non-drinker, moderate drinker, heavy drinker), smoking status (never smoker, past smoker, current smoker), and physical activity (low, moderate, high). ^2)^ Linear trends across categories of sugar-sweetened beverage consumption were tested using the median consumption values for each categories as an ordinal variable.
